# Myocardial arterial spin labeling in systole and diastole using flow‐sensitive alternating inversion recovery with parallel imaging and compressed sensing

**DOI:** 10.1002/nbm.4436

**Published:** 2020-11-04

**Authors:** Markus Henningsson, Carl‐Johan Carlhäll, Johan Kihlberg

**Affiliations:** ^1^ Unit for Cardiovascular Sciences, Department of Health, Medicine and Caring Sciences Linköping University Linköping Sweden; ^2^ Center for Medical Image Science and Visualization (CMIV) Linköping University Linköping Sweden; ^3^ School of Biomedical Engineering and Imaging Sciences King's College London London UK; ^4^ Department of Clinical Physiology in Linköping, Department of Health, Medicine and Caring Sciences Linköping University Linköping Sweden; ^5^ Department of Radiology, Department of Health, Medicine and Caring Sciences Linköping University Linköping Sweden

**Keywords:** acquisition, cardiovascular MR, compressed sensing, myocardial perfusion, perfusion and permeability, perfusion spin labeling

## Abstract

Quantitative myocardial perfusion can be achieved without contrast agents using flow‐sensitive alternating inversion recovery (FAIR) arterial spin labeling. However, FAIR has an intrinsically low sensitivity, which may be improved by mitigating the effects of physiological noise or by increasing the area of artifact‐free myocardium. The aim of this study was to investigate if systolic FAIR may increase the amount of analyzable myocardium compared with diastolic FAIR and its effect on physiological noise. Furthermore, we compare parallel imaging acceleration with a factor of 2 with compressed sensing acceleration with a factor of 3 for systolic FAIR. Twelve healthy subjects were scanned during rest on a 3 T scanner using diastolic FAIR with parallel imaging factor 2 (FAIR‐PI2_D_), systolic FAIR with the same acceleration (FAIR‐PI2_S_) and systolic FAIR with compressed sensing factor 3 (FAIR‐CS3_S_). The number of analyzable pixels in the myocardium, temporal signal‐to‐noise ratio (TSNR) and mean myocardial blood flow (MBF) were calculated for all methods. The number of analyzable pixels using FAIR‐CS3_S_ (663 ± 55) and FAIR‐PI2_S_ (671 ± 58) was significantly higher than for FAIR‐PI2_D_ (507 ± 82; *P* = .001 for both), while there was no significant difference between FAIR‐PI2_S_ and FAIR‐CS3_S_. The mean TSNR of the midventricular slice for FAIR‐PI2_D_ was 11.4 ± 3.9, similar to that of FAIR‐CS3_S,_ which was 11.0 ± 3.3, both considerably higher than for FAIR‐PI2_S,_ which was 8.4 ± 3.1 (*P* < .05 for both). Mean MBF was similar for all three methods. The use of compressed sensing accelerated systolic FAIR benefits from an increased number of analyzable myocardial pixels compared with diastolic FAIR without suffering from a TSNR penalty, unlike systolic FAIR with parallel imaging acceleration.

## Graphical abstract

Myocardial arterial spin labeling using flow‐sensitive alternating inversion recovery (FAIR) allows contrast‐free perfusion assessment but is hampered by an intrinsically low sensitivity. In this study we show that compressed sensing accelerated systolic FAIR benefits from an increased number of analyzable myocardial pixels compared with diastolic FAIR without suffering from increased physiological noise, unlike systolic FAIR with parallel imaging acceleration.

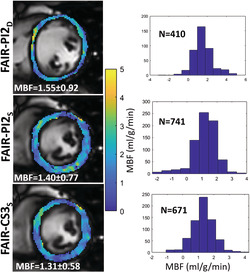

**Abbreviations used**
ANOVA: analysis of variance
ASL: arterial spin labeling
bSSFP: balanced steady‐state free precession
CS: compressed sensing
FAIR: flow‐sensitive alternating inversion recovery
FOV: field of view
MBF: myocardial blood flow
PI: parallel imaging
ROI: region of interest
SD: standard deviation
SENSE: sensitivity encoding
TSNR: temporal signal‐to‐noise ratio

## 1 INTRODUCTION

Arterial spin labeling (ASL) allows quantification of myocardial perfusion without the use of potentially toxic gadolinium‐based contrast agents, which are commonly used for first‐pass perfusion CMR.^1^ Exploiting intrinsic contrast mechanism enables repeated testing and perfusion evaluation in patients where contrast agents are contraindicated. Myocardial ASL involves “labeling” upstream arterial blood using radiofrequency pulses in the ascending aorta and proximal coronary arteries.^2^ Introducing a time delay between the labeling pulses and the image acquisition allows the labeled blood to reach the myocardium in the field of view (FOV), which yields a modulation in measured signal relative to the amount of blood received in the tissue. A second image can be acquired without labeling, which allows quantification of the tissue perfusion through subtraction of the two images, accounting for the T_1_ recovery during the delay period, and estimating the baseline M_0_ magnetization (typically by the acquisition of a third image without magnetization preparation).

An implementation of myocardial ASL, flow‐sensitive alternating inversion recovery (FAIR), involves the acquisition of two sets of magnetization‐prepared images: one “control” image preceded by a slice‐selective inversion pulse overlapping the FOV and one “tagged” image with a nonselective inversion pulse.^3^, ^4^, ^5^ An additional M_0_ image is also acquired to enable perfusion quantification. The image acquisition is limited to the middiastolic rest period to minimize cardiac motion. Furthermore, to achieve high perfusion sensitivity, a delay time of the order of 1 to 2 seconds is required. Although this may introduce cardiac motion‐related artifacts due to the difference in cardiac phase between the slice‐selective inversion pulse and the following image acquisition, this can be minimized by performing the inversion pulse in the same cardiac phase but in the preceding cardiac cycle relative to the image acquisition.^6^

A technical challenge of myocardial ASL is the intrinsically low sensitivity of the method due to the small percentage of tissue within each myocardial voxel that is subject to perfusion.^7^, ^8^ As a result, previous implementations of FAIR have employed strategies to improve the signal‐to‐noise ratio (SNR) of the measurements, including the use of 3 T scanners with balanced steady‐state free precession readout,^5^, ^9^, ^10^ multiple averages, image acceleration using parallel imaging (PI) to mitigate against noise from cardiac motion,^11^ and respiratory motion correction.^12^ Alternative ASL techniques have also been proposed to increase the sensitivity to myocardial perfusion.^13^, ^14^, ^15^, ^16^

In recent years, compressed sensing (CS)^17^ has emerged as a technique for image acceleration.^18^, ^19^ CS exploits the ability to compress the signal provided that it is sparse in some transformable domain, such as the wavelet domain, thus enabling data acquisition with high undersampling factors. In a previous study, PI acceleration was demonstrated to improve FAIR precision without affecting accuracy by shortening the data readout during the middiastolic rest period.^8^ Although the middiastolic rest period is typically longer than the end‐systolic rest period during resting conditions, systolic image acquisition would capture the myocardium at maximum thickness and thus may increase the number of analyzable pixels in the myocardium.^20^, ^21^, ^22^ Furthermore, systolic data acquisition would be preferable during stress perfusion when the diastolic quiescent duration shortens considerably.^23^ The aim of this study was to investigate if systolic FAIR may increase the amount of analyzable myocardium compared with diastolic FAIR and its effect on physiological noise. Furthermore, we compare PI acceleration with a factor of 2 with CS acceleration with a factor of 3 for systolic FAIR. The study was performed in 12 healthy subjects at rest.

## 2 METHODS

### 2.1 Cardiac FAIR pulse sequence and postprocessing

A previously published cardiac FAIR pulse sequence was implemented on a 3 T clinical scanner where six control, six tagged and one M_0_ image were used to reconstruct a single perfusion map.^6^ The image acquisition consisted of a balanced steady‐state free precession (bSSFP) readout. Furthermore, images were acquired in diastole and systole using PI with an acceleration factor of 2 (FAIR‐PI2_D_ and FAIR‐PI2_S_) and in systole using CS with an acceleration factor or 3 (FAIR‐CS3_S_). PI was performed with sensitivity encoding (SENSE), while the CS algorithm was a vendor‐provided, wavelet‐based algorithm with inline reconstruction.^17^ The imaging parameters for the different imaging strategies are shown in Table 1. A 30 mm slice thickness was used for the inversion pulse of the control images while the tagged images were acquired with a nonselective inversion pulse. All images were acquired during breath‐holding where one pair of tagged and control images were acquired in a breath‐hold and spaced ~8 seconds apart. The order of tagged and control images alternated for each breath‐hold to minimize potential bias due to incomplete M_z_ recovery. The FAIR pulse sequence was double‐gated, where both inversion pulses and image acquisitions were triggered to the cardiac rest period in adjacent cardiac cycles. Similar to previously published studies using double‐gating, the center of k‐space for the image acquisition and the inversion pulses had the same trigger delay.^4^, ^6^ The M_0_ image was acquired in a separate scan without any contrast preparation to estimate the baseline magnetization.

**TABLE 1:** Imaging parameters

	FAIR‐PI2_D_	FAIR‐PI2_S_	FAIR‐CS3_S_
Cardiac phase	Diastole	Systole	Systole
Acceleration method	SENSE	SENSE	Compressed sensing
Acceleration factor	2	2	3
Flip angle (°)	50	50	50
Ramp‐up pulses	20	20	25
FOV (mm)	300 × 300	300 × 300	300 × 300
Pixel bandwidth (Hz)	1890	1890	1890
Δx (mm^3^)	2 × 2 × 10	2 × 2 × 10	2 × 2 × 10
Acquisition matrix	150 × 150	150 × 150	150 × 150
TR (ms)	2.2	2.2	2.2
TE (ms)	1.1	1.1	1.1
T_acq_ (ms)	165	165	110

**FIGURE 1:**
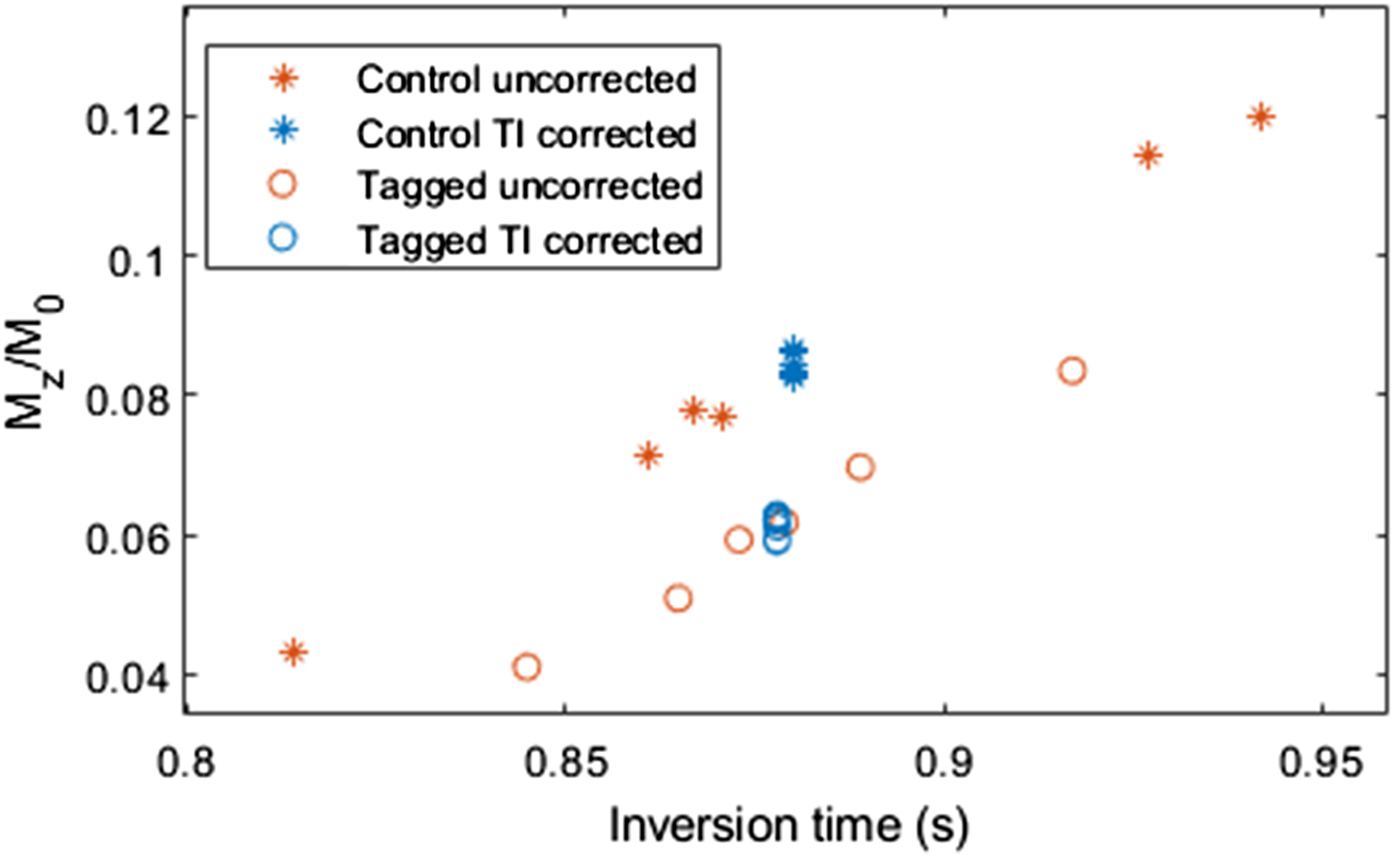
Measured myocardial signal intensities for control (stars) and tagged (dots) images before (red) and after (blue) correction for differences in inversion times for the six sets of images. The inversion correction shifts the data to the same time point (mean inversion time) using an inversion recovery signal model, prior to averaging of the control and tagged images. The differences in mean inversion times between tagged and control images cause the corrected data to be at slightly different inversion times, which can be accounted for during the MBF calculation, as defined in Equation 2

**FIGURE 2:**
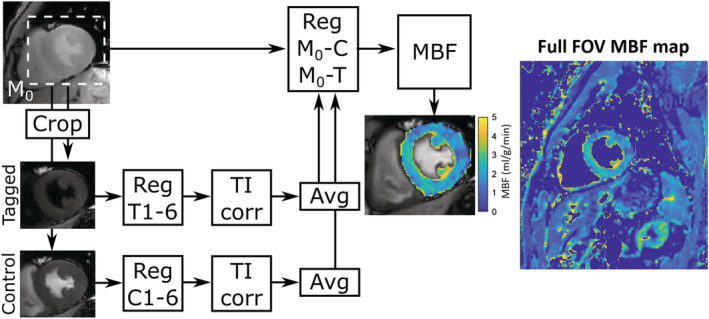
Schematics of the myocardial FAIR postprocessing. A region of interest is cropped around the left ventricle in short‐axis using the M_0_ image. The cropped region is applied to the six control and tagged FAIR images. Images 2‐6 are then registered to image 1 (REG T1–6/C1–6) for both the tagged and control images to minimize motion within each series. To account for differences in inversion times, the signal intensities are then modulated based on the inversion recovery signal model (TI corr). The six images are then averaged (Avg), yielding one tagged and one control image, which are registered to the cropped M_0_ image (REG M_0_‐C M_0_‐T). Finally, myocardial blood flow maps are calculated using Equation 2. A full FOV MBF map from the same subject and with the same color scale is shown on the right

To account for any differences in positions between breath‐holds and different image types, rigid body (translation and rotation) image registration was performed first for the control and tagged images separately, followed by rigid body correction between the image types. The image registration was implemented using the MATLAB (MathWorks, Natick, MA, USA) function imregister. First, a region of interest (ROI) for motion tracking was selected around the left ventricle in the M_0_ image and propagated to all tagged and control images. Then all cropped tagged and control images were registered to the first image in their respective time series using a mean squares similarity metric. To allow for correction of differences in inversion times during the scans, the actual inversion times were stored for all images. The data were corrected using the formula:

(1) ITIcorr=M0+I−M0eΔTIT1,

where *I* is the signal intensity in the target or control images, *ΔTI* is the difference between actual and nominal (average for all six images) inversion time and *T*_1_ is the longitudinal relaxation time of blood of ~1700 ms.^24^ Measured signal intensities for the six tagged and control images before and after inversion time correction are shown in Figure 1. Following the inversion time correction, a single tagged and control image were obtained by averaging across the series. The resulting images were then registered to the M_0_ image using a mutual information rigid body registration to account for any respiratory motion between images. Finally, myocardial blood flow (MBF) was calculated using the formula for double‐gated myocardial ASL^4^, ^6^:

(2) $$\mathrm{MBF}=\frac{1}{2M_{0}}\left(\frac{C}{{\mathrm{TI}}_{C}\cdot e^{\frac{-{\mathrm{TI}}_{C}}{T_{1}}}}-\frac{T}{{\mathrm{TI}}_{T}\cdot e^{\frac{-{\mathrm{TI}}_{T}}{T_{1}}}}\right),$$

where *C* and *T* are the control and tagged images, respectively. Note that separate inversion times were used for the tagged and control images, based on the mean inversion time from the *T*_1_ correction using Equation 1. The FAIR postprocessing pipeline is illustrated in Figure 2.

### 2.2 MRI experiments

This study was approved by the regional ethics committee. Twelve healthy subjects (age: 29.4 ± 3.9 years; eight males and four females) were recruited and provided written informed consent. All 12 subjects had no history of prior or current cardiovascular disease or medication. All experiments were performed on a 3 T clinical scanner (Achieva, Philips Healthcare, Best, the Netherlands) with a 24‐channel torso coil. To compare the three myocardial FAIR pulse sequences, a midventricular slice was acquired during rest in short‐axis with FAIR‐PI2_D_, FAIR‐PI2_S_ and FAIR‐CS3_S_ in a randomized order. Assuming a heart rate of 60 bpm, a set of FAIR (tagged and control) images were acquired in 12 seconds, including an 8‐second rest period to allow for M_z_ recovery. The FAIR scan was repeated six times for each method to generate the same amount of signal averages. For each repetition, the order of control and tagged images was alternated to eliminate bias. No dietary restrictions (eg, caffeine) were stipulated for the volunteers prior to participation. All scans were ECG‐triggered and the systolic and diastolic rest periods were visually determined from a four‐chamber cine image with a temporal resolution of 15 ms.

### 2.3 Image analysis and statistics

The reconstructed images were transferred to an offline workstation for postprocessing using MATLAB, as outlined in Figure 2. The myocardium of the left ventricle was manually segmented, and perfusion was calculated as the mean MBF (in milliliters per grams per minute) in the segmented ROI. The number of pixels included in the segmented myocardium was recorded for all MBF maps. To estimate the effects of cardiac motion, physiological noise was estimated by calculating MBF maps for each control‐tagged image pair and dividing the mean MBF with the standard deviation (SD) across the six maps. This metric was first described by Poncelet et al and is typically referred to as temporal SNR (TSNR).^4^ The TSNR and mean MBF were calculated for FAIR‐PI2_D_, FAIR‐PI2_S_ and FAIR‐CS3_S._ Differences were compared using a one‐way analysis of variance (ANOVA). A threshold of *P* less than .05 was used to reject the null hypothesis that the methods yield the same TSNR or mean MBF. If the null hypothesis was rejected, posthoc t‐tests were performed to determine which groups were statistically different with a significance threshold of *P* less than .05.

## 3 RESULTS

**FIGURE 3:**
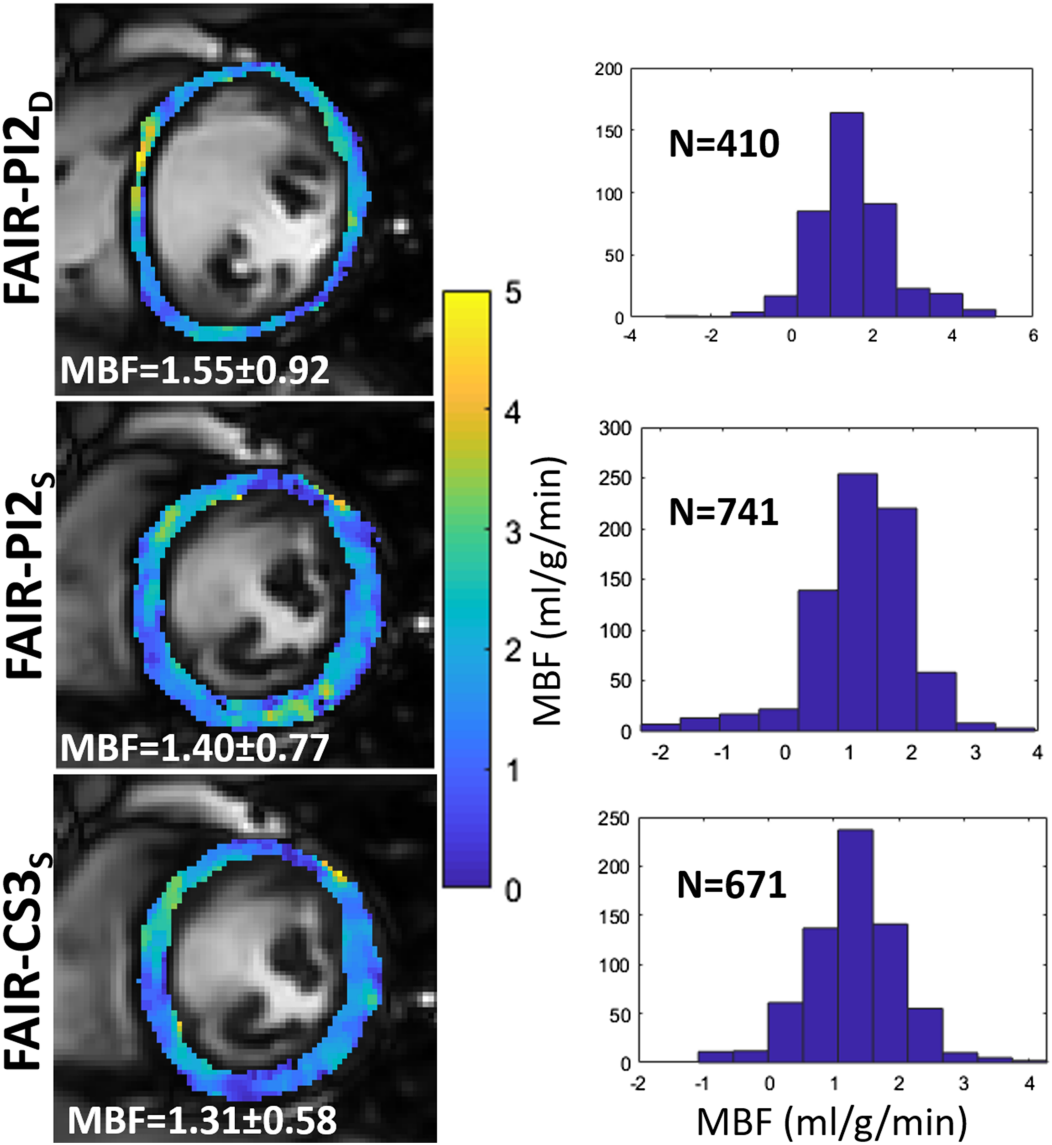
Manually segmented perfusion maps obtained using FAIR‐PI2_D_, FAIR‐PI2_S_ and FAIR‐CS3_S_ and corresponding histograms of myocardial pixels. Due to the cardiac contraction the myocardium becomes thicker in the systolic images, yielding more analyzable pixels (N) compared with the diastolic image

**FIGURE 4:**
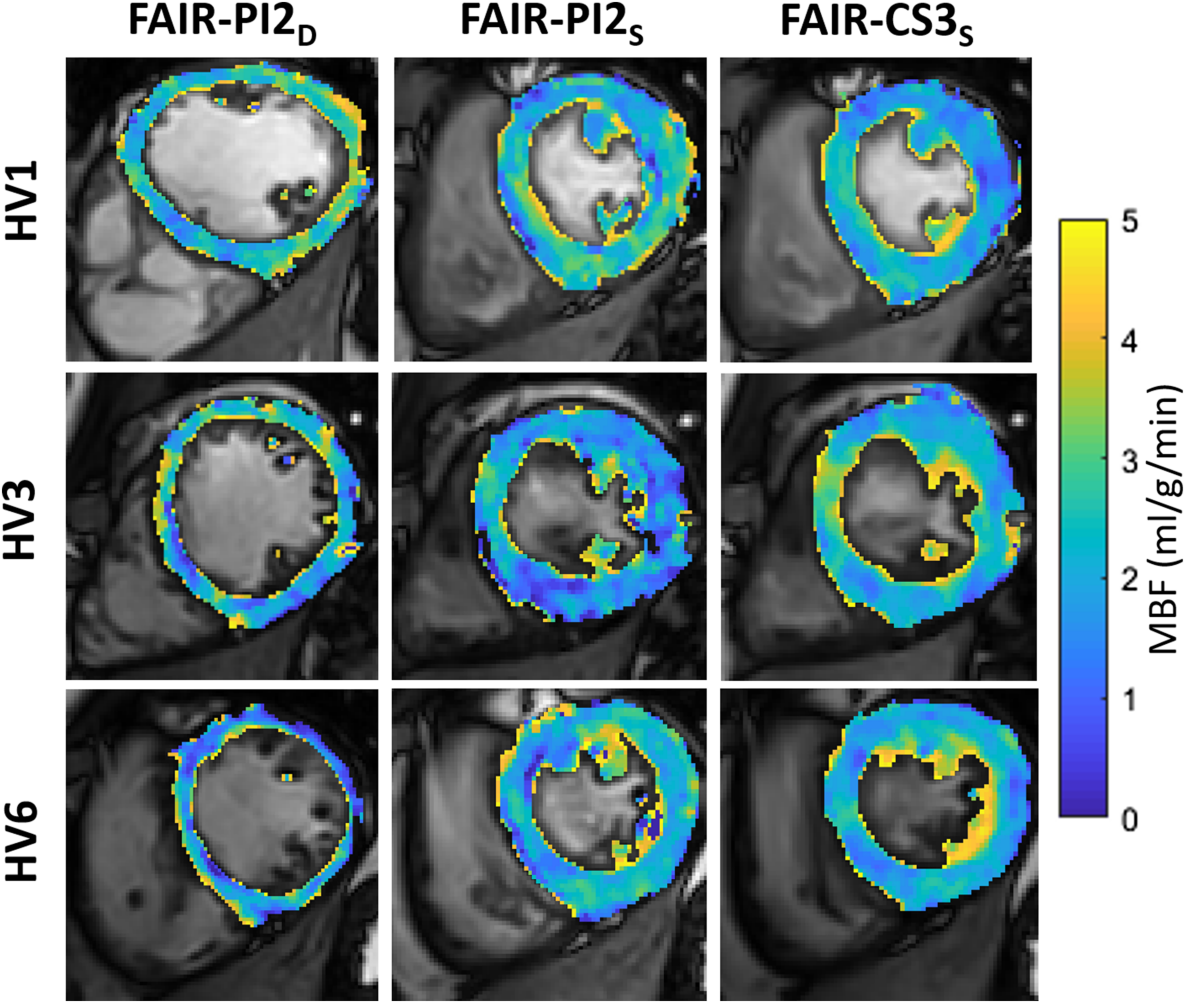
Perfusion maps obtained in three healthy volunteers (HV1, HV3 and HV6) using diastolic FAIR with parallel imaging factor 2 (FAIR‐PI2_D_) and systolic FAIR with parallel imaging factor 2 (FAIR‐PI2_S_) and compressed sensing factor 3 (FAIR‐CS3_S_)

The mean heart rate ± SD for the 12 healthy volunteers during rest was 64 ± 10 bpm. The average number of pixels ± SD in the segmented myocardium for FAIR‐PI2_D_ was 507 ± 82, significantly less than for FAIR‐PI2_S,_ which was 671 ± 58 (*P* = .001). Similarly, the number of segmented pixels using FAIR‐CS3_S_ (663 ± 55) was significantly higher than for FAIR‐PI2_D_ (*P* = .001), while there was no significant difference between FAIR‐PI2_S_ and FAIR‐CS3_S_. Representative segmentations of the left ventricular myocardium, fused onto the raw control images, are shown in Figure 3, which also shows the MBF histograms from the segmentation for all three techniques. Representative perfusion maps from three healthy volunteers are shown in Figure 4, demonstrating comparable perfusion values and similar image quality using systolic and diastolic FAIR with PI2 and CS3 acceleration.

**FIGURE 5:**
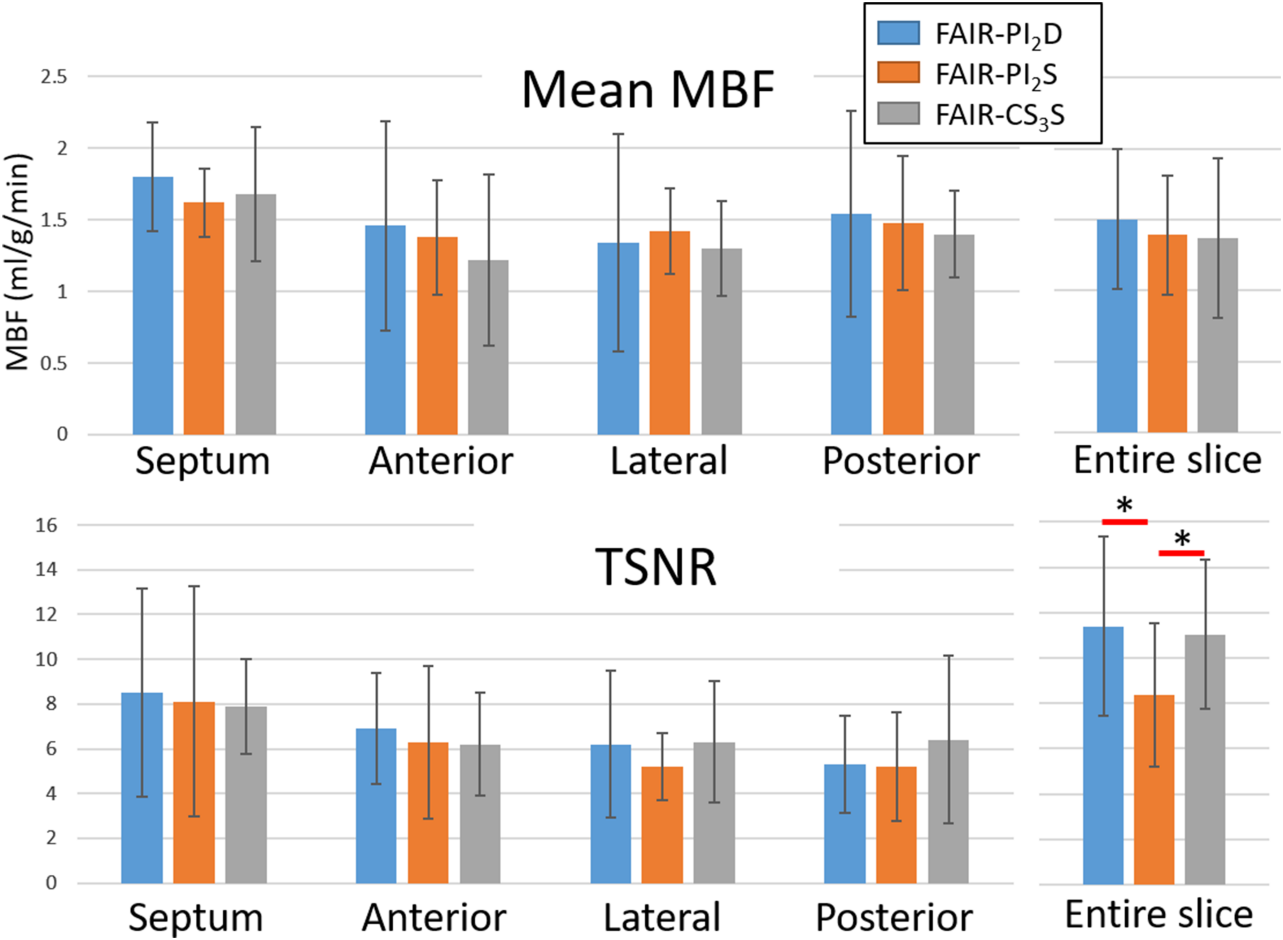
Temporal signal‐to‐noise ratio (TSNR) and mean myocardial blood flow (MBF) values for all 12 healthy volunteers using FAIR‐PI2_D_, FAIR‐PI2_S_ and FAIR‐CS3_S_. Values are presented both for regional (septum, anterior, lateral and posterior) measurements and for the midventricular short‐axis entire slice. * denotes statistically significant differences

The mean MBF and TSNR for the three techniques in the midventricular short‐axis slice for all 12 healthy subjects are summarized in Figure 5. The group‐wise mean ± SD of the mean MBF (in ml/g/min) for the entire midventricular slice for FAIR‐PI2_D_, FAIR‐PI2_S_ and FAIR‐CS3_S_ was 1.5 ± 0.5, 1.4 ± 0.4 and 1.4 ± 0.6, respectively. Similar mean MBF were measured for all three techniques for both regional measurements and for the entire slice. The ANOVA test showed that there were no statistically significant differences in mean MBF between any groups. The mean TSNR of the midventricular slice for FAIR‐PI2_D_, FAIR‐PI2_S_ and FAIR‐CS3_S_ was 11.4 ± 3.9, 8.4 ± 3.1 and 11.0 ± 3.3, respectively. The mean TSNR difference between FAIR‐PI2_D_ and FAIR‐PI2_S_ was statistically different (*P* < .05), as was the difference between FAIR‐CS3_S_ and FAIR‐PI2_S_ (*P* < .05). However, the regional TSNR measurements did not yield any statistically significant differences between FAIR‐PI2_D_, FAIR‐CS3_S_ and FAIR‐PI2_D_.

## 4 DISCUSSION

In this work we have investigated the merits of systolic image acquisition to increase the amount of analyzable myocardium of FAIR ASL. Furthermore, we have compared conventional PI acceleration with a factor of 2 with CS with a factor of 3 for systolic FAIR. We found that systolic imaging appears to be advantageous for myocardial FAIR due to the increased myocardial thickness during this cardiac phase, which yields fewer partial volume effects compared with conventional diastolic imaging. This leads to a higher number of pixels in the analyzable myocardium. Furthermore, the use of CS appears to reduce the amount of physiological noise in the MBF maps, as measured using TSNR, to a similar level as the diastolic MBF maps acquired with PI. The amount of physiological noise of the estimated perfusion is one of the main limitations of noncontrast myocardial perfusion techniques, and the advantages provided by systolic imaging using CS may improve the sensitivity of myocardial FAIR to detect perfusion defects in patients.

The merits of systolic imaging have also been noted in a similar quantitative cardiac application, namely, myocardial T_1_ mapping.^20^, ^21^, ^22^ Similar to myocardial FAIR, T_1_ mapping is based on single‐shot acquisitions whose duration may extend beyond the quiescent period of the systolic rest period. Notwithstanding this limitation, systolic imaging appears to be advantageous for these applications. As noted in previous work on T_1_ mapping, systolic imaging benefits from robustness to high heart rates and tachyarrhythmia.^23^ The end‐systolic rest period is relatively invariant to changes in heart rate while the middiastolic rest period is strongly correlated with the heart rate.^25^ When the cardiac cycle is short, due to stress or arrhythmia, the diastolic quiescent period may become shorter than the systolic rest period or almost disappear entirely. Therefore, systolic imaging may be particularly beneficial to enable robust, high‐quality myocardial FAIR acquisition during stress testing or in the event of sustained arrhythmia.

As noted in previous studies, accelerating the image acquisition reduces the detrimental effects of physiological noise due to cardiac motion.^5^, ^11^ Cardiac motion may be even more significant during the systolic compared with the diastolic phase, leading to increased physiological noise. Using PI the acquisition time was 165 ms per image, which is typically much longer than the systolic quiescent period. We found a significant reduction in TSNR for FAIR‐PI2_S_ compared with FAIR‐PI2_D_, which indicates an increased amount of cardiac motion in the systolic acquisition. However, by accelerating the systolic data acquisition to 110 ms using CS, the TSNR was increased to a comparable level with the diastolic maps, which suggests that the systolic cardiac motion had been effectively minimized. A recent study comparing diastolic and systolic cardiac FAIR using PI acceleration found a similar reduction in TSNR using systolic compared with diastolic FAIR, while the global MBF was lower than with diastolic acquisition.^26^ In the current study, we did not find any mean differences in global MBF between systole and diastole. Differences in acquisition strategies may explain these discrepancies: a shorter acquisition window (165 ms for PI_2_) and ECG triggering were used in the current study while Javed et al used an acquisition window of 192 ms and fingertip plethysmograph triggering.^26^ The reduction in acquisition time in our study is largely due to an ~30% shorter repetition time of 2.2 ms compared with 3.1 ms in Javed et al.^26^ This is a significant advantage as it enables more efficient data acquisition, translating into a shorter acquisition window and/or higher spatial resolution. However, further reducing the acquisition time will be important for systolic FAIR due to the higher likelihood of cardiac motion. To this end, advanced image acceleration techniques such as CS hold great promise to reduce the physiological noise for systolic perfusion images where the acquisition time may approach the duration of the systolic rest period of ~80‐100 ms. In the current study, we used a vendor‐provided wavelet‐based CS technique.^13^ Alternative CS techniques using total variation,^27^ low‐rank schemes,^28^ adaptive sparsity transform generation^29^, ^30^ or new deep learning techniques^31^, ^32^ may allow acceleration with higher factors to further reduce the physiological noise. However, it should be noted that CS intrinsically removes some noise during the iterative thresholding procedure. Although it is beyond the scope of this study to investigate how different CS parameters influence the MBF noise profile, it is important to recognize that this reconstruction approach may disguise subtle local perfusion changes. However, in this study we observed slight changes between different regions of the myocardium, consistent with previous studies where higher MBF was found in the septum.^13^ This variation was found for both CS and conventional PI suggesting that comparable changes caused by perfusion defects may be detectable using CS.

The midventricular group mean MBF values obtained with the implemented cardiac FAIR method of ~1.5 ml/g/min are comparable, but at the higher end, relative to previous studies using the same technique, which range from 1.0 to 1.5 ml/g/min.^5^, ^9^, ^11^, ^12^, ^13^, ^16^ Notable differences compared with previous implementations of cardiac FAIR are a shorter repetition time (2.2 ms here compared with ~3.2‐4 ms in previous studies), the absence of fat suppression prepulse and potentially different postprocessing pipelines. In the current study, we performed inversion time correction on the six sets of control and tagged images prior to averaging, motion correction and MBF calculation. Alternatively, MBF may be calculated for each set of images (one MBF map for each breath‐hold) and then averaged. This would avoid the need for inversion time correction as the recorded inversion times for all images would be directly applied to Equation 2. However, the drawback with this approach is that motion correction is required between the tagged and control images six separate times and is more likely to introduce registration errors due to the substantial difference in contrast to between the noisy images. By contrast, with the proposed approach, the six different images with roughly the same contrast are registered to each other with minimal error then averaged to yield higher quality tagged and control images prior to the single mutual information registration. Further work is required to optimize the MBF postprocessing and improve respiratory motion correction. A free‐breathing cardiac FAIR would be desirable, which would facilitate patient studies but increase the necessity for robust and accurate motion correction.^12^, ^13^

We found similar mean MBF values for systolic and diastolic FAIR. This contrasts with findings from coronary flow measurements where peak blood flow typically occurs during diastole when the myocardium relaxes, increasing the pressure gradient between the aortic root and coronary arteries.^33^ Based on the observation that coronary blood flow is higher in diastole than systole, a similar increase in myocardial perfusion could be expected during the diastolic phase. Studies using quantitative contrast‐enhanced myocardial perfusion techniques have found similar MBF during rest while higher MBF were found for diastolic acquisitions during stress.^34^, ^35^ However, it should be noted that ASL techniques such as myocardial FAIR rely on perfusion‐sensitizing inversion pulse in the same cardiac phase but preceding cardiac cycle as the image acquisition. Therefore, the measured MBF using myocardial FAIR reflects the integration of perfusion across the entire cardiac cycle, and the systolic and diastolic MBF should in theory be the same, even during stress. Further studies during stress conditions are required to verify this claim.

In conclusion, systolic imaging allows cardiac FAIR acquisitions with larger analyzable myocardium compared with diastolic imaging. Furthermore, the use of CS‐accelerated systolic FAIR does not suffer from a TSNR penalty, unlike systolic FAIR with PI acceleration. Systolic imaging is particularly useful during stress testing as it is more stable in timing, both in terms of trigger delay and rest period duration, while the diastolic rest period may become significantly shortened.
